# Supplementary material for: Smoking prevalence, core/periphery network positions, and peer influence: Findings from five datasets on US adolescents and young adults
Source: PLoS One. 2021 Mar 24;16(3):e0248990. doi: 10.1371/journal.pone.0248990 (PMC7990186; doi:10.1371/journal.pone.0248990)
Supplement: Attachment — Submitted filename: PlosOne_Comments_Nov20.docx [file pone.0248990.s002.docx]

"Smoking prevalence, core/periphery network positions, and peer influence: findings from five datasets on US adolescents."

This is an interesting paper that addresses a gap in the evidence base. The conceptual idea has been well thought out and the execution of the analytical methods are ‘technically rigorous’, as required by PLOS ONE. However, I do have concerns regarding drawing conclusions from the datasets that vary considerably in demographic characteristics (in particular, age), and most importantly, the social network that has been measured. Finally, the results section is generally erratic and confusing to follow, and would benefit from refining it to be more concise and present the main findings to the reader.

**General comments**

1. Careful spellcheck required, alongside a grammar check (several sentences begin with ‘And’).
2. Requires consistency in the reference to schools – you both name them individually or refer to them as sample datasets 1-5.

**Introduction**

3. Page 4: Social networks should be introduced prior to outlining the hypotheses. You assume that the reader has prior knowledge of social network terminology, but do not define or explain social network concepts (i.e. network positions and peer influence processes).
4. Page 5: The paragraph outlining minority influence is confusing – can you explain what you mean by ‘it is assumed the majority and minority can both be influential’. The terms ‘minority’ and ‘majority’ should be defined.
5. Page 6: consider re-phrasing the term ‘disclose the black box in previous research’

**Materials and methods**

6. Page 6: ‘The students in participating schools were asked to report their smoking frequency in the past 30 days..’ – this information is repeated on page 8 ‘Smoking Measures’
7. Page 8: You do not provide any information on the social network questions included in the NetSense and NetHealth datasets. You cannot make comparisons across different types of networks, only if they too are friendship networks (to be consistent with Add Health networks).
8. Page 9: Analytical strategy – given the demographic variance in your datasets, why have you not controlled for characteristics such as parental education and race?

**Results**

9. Page 11: the results section is generally hard to follow as you report a lot of results but do not explain them in detail. What relevance is there in reporting the cross-sectional and t-test results? It is difficult to follow all of the results as they are erratic and confusing – you report results from some schools and not others. In general, it is my recommendation that the results section could be refined to be more concise.
10. Page 13: ‘Regarding the rest effects in the behaviour equation’ – can you clarify what this means?
11. Page 13: ‘in the network equation, the rate parameters suggest that adolescents had about 15, 10, 9 11 and 26 opportunities…’ – it is unclear what you mean by this statement.

**Discussion**

12. Page 16-17: ‘we have no access to these two …but there is a good chance’ – you should not make inferences in this case
13. Page 17: Only in the discussion do you address the disparity of the social network questions and the age range of participants. The Add Health study measures a completely different network to the NetSense and NetHealth studies – this is highly important and raises the question of how representative the results are when you have two different networks. Furthermore, whilst you address the difference in age (Add Health participants are young adolescents compared to late adolescents/emerging adults in NetSense and NetHealth) you do not address the time frame. It is important to consider the implications of the different periods in time that the studies were set.
