# Supplementary material for: Smoking prevalence, core/periphery network positions, and peer influence: Findings from five datasets on US adolescents and young adults
Source: PLoS One. 2021 Mar 24;16(3):e0248990. doi: 10.1371/journal.pone.0248990 (PMC7990186; doi:10.1371/journal.pone.0248990)
Supplement: Attachment — Submitted filename: Response to Reviewers.docx [file pone.0248990.s003.docx]

**Response to Reviewers**

PONE-D-20-32056

Smoking prevalence, core/periphery network positions, and peer influence: findings from five datasets on US adolescents

PLOS ONE

I would like to thank the editor and reviewers for their very constructive feedback. Incorporating the reviewers’ suggestions for revision has resulted in a greatly improved manuscript. Below, I note the concerns of the reviewers and then explain how I responded to each comment. In all cases, reviewer comments are in italics, while my responses are in plain text.

**Reviewer 1**

*1. Careful spellcheck required, alongside a grammar check (several sentences begin with ‘And’).*

––––––––––––––––––––––––––––––––––––––––––––––––––––

I thank the reviewer for this suggestion. I have conducted a thorough spellcheck and grammar check, with all sentences that begin with “And” revised.

*2.* *Requires consistency in the reference to schools – you both name them individually or refer to them as sample datasets 1-5.*

––––––––––––––––––––––––––––––––––––––––––––––––––––

This is a valid point. In the revised manuscript I consistently refer to them as sample datasets 1-5.

*3. Page 4: Social networks should be introduced prior to outlining the hypotheses. You assume that the reader has prior knowledge of social network terminology, but do not define or explain social network concepts (i.e. network positions and peer influence processes).*

––––––––––––––––––––––––––––––––––––––––––––––––––––

This is a critical comment. Members occupying core positions form a central and densely connected cluster in a social network, and those occupying peripheral positions of a network have just a few links or no connections at all. I now explain these two network position concepts in the second paragraph on page 3 in the revised manuscript.

Peer influence refers to the propensity of a person to alter his or her behavior or attitude such that it becomes more similar to that of his or her contacts. I now explain this concept in the last paragraph on page 4 in the revised manuscript.

*4. Page 5: The paragraph outlining minority influence is confusing – can you explain what you mean by ‘it is assumed the majority and minority can both be influential’. The terms ‘minority’ and ‘majority’ should be defined.*

––––––––––––––––––––––––––––––––––––––––––––––––––––

Majority is the greater (more than half) subset of individuals and minority is the smaller (less than half) subset of individuals in a population. I now clarify these two terms in the second paragraph on page 5 in the revised manuscript.

*5. Page 6: consider re-phrasing the term ‘disclose the black box in previous research’.*

––––––––––––––––––––––––––––––––––––––––––––––––––––

I revise this sentence as “in this study we aim to extend this line of research with several empirical datasets” on page 6.

*6. Page 6: ‘The students in participating schools were asked to report their smoking frequency in the past 30 days.’ – this information is repeated on page 8 ‘Smoking Measures’.*

––––––––––––––––––––––––––––––––––––––––––––––––––––

I remove the duplicated part and revise this sentence as “The students in participating schools had an average smoking prevalence of 25%” on page 6.

*7. Page 8: You do not provide any information on the social network questions included in the NetSense and NetHealth datasets. You cannot make comparisons across different types of networks, only if they too are friendship networks (to be consistent with Add Health networks).*

––––––––––––––––––––––––––––––––––––––––––––––––––––

I am sorry for the confusion. The reviewer is right that NetSense and NetHealth project used a different strategy from the Add Health study to collect information on friendship networks. The Add Health study used the “name generator”, i.e., a question for the participants to nominate up to five female and five male friends, as indicated on page 7. Instead, the NetSense and NetHealth projects built friendship networks among their participants with real-word communication events that were aggregated from the call detailed records (CDRs) retrieved from the participants’ smartphones, as indicated on page 8.

*8. Page 9: Analytical strategy – given the demographic variance in your datasets, why have you not controlled for characteristics such as parental education and race?*

––––––––––––––––––––––––––––––––––––––––––––––––––––

I now clarify on page 10 that “Given the demographic variance in the five sample dataset, we also control for covariates such as gender, age, parental education, and race in the SAB models”.

*9. Page 11: the results section is generally hard to follow as you report a lot of results but do not explain them in detail. What relevance is there in reporting the cross-sectional and t-test results? It is difficult to follow all of the results as they are erratic and confusing – you report results from some schools and not others. In general, it is my recommendation that the results section could be refined to be more concise.*

––––––––––––––––––––––––––––––––––––––––––––––––––––

I follow the reviewer’s suggestion and remove the *t*-test part from the “Results” section in the revised manuscript.

*10. Page 13: ‘Regarding the rest effects in the behaviour equation’ – can you clarify what this means?*

––––––––––––––––––––––––––––––––––––––––––––––––––––

I revise it to “Regarding the rest of the effects in the behavior equation”.

*11. Page 13: ‘in the network equation, the rate parameters suggest that adolescents had about 15, 10, 9 11 and 26 opportunities…’ – it is unclear what you mean by this statement.*

––––––––––––––––––––––––––––––––––––––––––––––––––––

I am sorry for the confusion. I now revise it as “In the network equation, the rate parameters suggest that the estimated numbers of opportunities for change in network ties (i.e., -1 tie, no change, +1 tie) per adolescent between two observation time points are about 15, 10, 9, 11, and 26 in the five cohorts, respectively”.

*12. Page 16-17: ‘we have no access to these two …but there is a good chance’ – you should not make inferences in this case.*

––––––––––––––––––––––––––––––––––––––––––––––––––––

I revise it to “We have no access to these two datasets. Future research is needed to examine the findings from the current study in more robust and generalizable settings.”

*13. Page 17: Only in the discussion do you address the disparity of the social network questions and the age range of participants. The Add Health study measures a completely different network to the NetSense and NetHealth studies – this is highly important and raises the question of how representative the results are when you have two different networks. Furthermore, whilst you address the difference in age (Add Health participants are young adolescents compared to late adolescents/emerging adults in NetSense and NetHealth) you do not address the time frame. It is important to consider the implications of the different periods in time that the studies were set.*

––––––––––––––––––––––––––––––––––––––––––––––––––––

I totally agree with the reviewer there are various types of disparities across the Add Health, NetSense, and NetHealth studies. In the manuscript I honestly discussed their differences in how social networks were constructed, age composition of participants, and time frame of each sample dataset. I also controlled the demographic variance in the five sample dataset, including covariates such as gender, age, parental education, and race in the SEM analyses and SAB models.

Having said that, we lack nationally representative samples continually covering US adolescents in the past 30 years that allow us to investigate the evolutionary patterns of smoking prevalence, network positions, and peer influence with aforementioned disparities fully considered. The Add Health data were collected in 1995 and 1996, the NetSense data were collected in 2011 and 2012, and the NetHealth data were collected in 2015 and 2016. In this study there is no dataset from the first decade of the 21st century. I have contacted the authors of Mathys et al. (2013) but got no response. While PROSPER data are available for researchers at Penn State University, limited accessibility is provided for scholars outside Penn State. Due to the constraint in data availability, I am very cautious and not claiming that I am examining the changing patterns of US adolescents from different periods in time. Therefore, the title of this manuscript is “Smoking prevalence, core/periphery network positions, and peer influence: **findings from five datasets on US adolescents**”.

To sum up, this study aims to present some common patterns of the five sample datasets, with various types of disparities acknowledged. I expect these patterns to be confirmed or challenged by collaboration of scientists across a wide range of disciplines in the near future.

**Reviewer 2**

*1. In the introduction and abstract the author writes as if the paper is about adolescents, but it is actually about adolescents and young adults. Therefore, both literatures should be incorporated.*

––––––––––––––––––––––––––––––––––––––––––––––––––––

We thank the reviewer for this suggestion. As shown in Table 1, the participants had a range of age between 14-18 in sample dataset 1, 12-19 in sample dataset 2, 11-18 in sample dataset 3, 17-19 in sample dataset 4, and 17-20 in sample dataset 5.

The US Food and Drug Administration (FDA) defines adolescents as individuals in the 12-21 years age group (FDA 2003). American Academy of Pediatrics (AAP) identifies adolescence as 11 to 21 years of age, including early (ages 11-14 years), middle (ages 15-17 years), and late (ages 18-21years) adolescence (Hagan et al. 2008).

Therefore, I consider the participants in the five sample datasets as adolescents. But I clarify the FDA and AAP’s definitions of adolescents on pages 10-11 in the revised manuscript when introducing the age composition of the participants in Table 1.

*2. There are some points of clarification that would help in the methods. First, the authors clearly elucidate a data collection issue that they are avoiding based on a cutoff. Three schools were chosen. How many schools met the 85% cutoff? What additional criteria were applied to select amongst those or were there only 3 available schools?*

––––––––––––––––––––––––––––––––––––––––––––––––––––

This is a great question. After excluding school 175, only three out of fifteen saturated schools – Jefferson High, Sunshine High, and school 28 – met the 85% cutoff.

As we introduced in the manuscript, the Add Healthy study suffered from an administrative error, i.e., some students were only allowed to ask one female and one male best friends during the wave 1 In-Home survey. While missing data is a well-known challenge for traditional cross-sectional and longitudinal studies, the problem is even greater for network studies. As indicated in Handcock (2002), if we lack information on *f* participants in a network, the fraction of unobserved edges will be 1-(1-*f*)^2^. The following table shows four schools with the lowest proportion of limited nomination. In school 28, about 14% adolescents encountered the limited nomination issues and 27% edges were unobserved; but in school 88, about 26% adolescents encountered the limited nomination issue and 45% edges were unobserved. Therefore, we set cutoff at 85% and only pick three schools from the Add Healthy study.

| Add Health School | % limited nomination (*f*) | % unobserved edges (1-(1-*f*)^2^) |
| --- | --- | --- |
| Sunshine High | 5.23% | 10.19% |
| Jefferson High | 5.71% | 11.09% |
| 28 | 14.40% | 26.72% |
| 88 | 26.03% | 45.28% |

*3.* *In the methods you introduce a variety of analyses and strategies. Each of these should include a clear statement about why it was chosen, where it has been used before and which hypothesis it is linked to. This will help later as you go through the very long results section.*

––––––––––––––––––––––––––––––––––––––––––––––––––––

This is a crucial comment. To test the first and second hypotheses, I estimate a structural equation model (SEM) for each sample dataset using Stata V16.0 because we have multiple causes and multiple outcomes simultaneously. The SEM analysis is appropriate for longitudinal study of the multiple-path relationships between adolescent peer networks and their smoking behavior (Hall & Valente 2007; Lakon & Hipp 2014).

To test the third hypothesis, I adopt the Stochastic Actor-Based (SAB) modeling strategy implemented in RSiena. The SAB modeling strategy has been widely used to investigate the peer influence via social networks on adolescent smoking behavior in recent years (Shoham et al. 2012; Schaefer et al. 2012; Mathys et al. 2013; Haas & Schaefer 2014; Lakon et al. 2014; Osgood et al. 2015; Wang et al. 2016, 2018; Montgomery et al. 2020). The average similarity effect indicates whether peer influence exists in an environment or not. The creation function is used to measure the magnitude of peer influence on increasing smoking levels and the endowment function is used to measure the magnitude of peer influence on decreasing smoking levels (Haas & Schaefer 2014).

I now clarify these details under the subheader “Analytical Strategy” in “Materials and methods” section on pages 9-10.

*4.* *Then, when you get into the results, it might be helpful to organize them by hypotheses explicitly instead of approach. I understand why you have it organized the way you do, but there’s a lot going on and it’s easy to get lost in the weeds of the results section.*

––––––––––––––––––––––––––––––––––––––––––––––––––––

I now follow the reviewer’s suggestion and revise the subheaders to “Hypotheses I & II: SEM Results” on page 11 and “Hypothesis III: Findings from SAB Models” on page 12 in the “Results” section.

*5. A note about the results and discussion, you switch between calling the Notre Dame date University of Notre or naming it by the two dataset contained therein. Pick one and stick with it.*

––––––––––––––––––––––––––––––––––––––––––––––––––––

As the reviewer has suggested, I stick to sample datasets 4 and 5 in the “Results” and “Discussion” section in the revised manuscript.

*6. Page 17, in the T-test results section, there are a number of sentences with words missing (e.g. Regarding the rest network statistics).*

––––––––––––––––––––––––––––––––––––––––––––––––––––

I follow the suggestion from reviewer 1 and remove the *t*-test part from the “Results” section in the revised manuscript.

*7. On page 12, the following sentence should be rewritten from, “The peer influence measured as average” to “The peer influence was measured as an average”.*

––––––––––––––––––––––––––––––––––––––––––––––––––––

I revise this sentence on page 12 as the reviewer suggested.

*8. On page 12, the following sentence is missing a awkward, “the result of which matches that in previous studies of these two schools.” It should be reworded as, “the result of which matches previous studies of these two schools”.*

––––––––––––––––––––––––––––––––––––––––––––––––––––

I revise this sentence on page 12 as the reviewer suggested.

*9. One page 13, the following phrase, “Regarding the rest effects in the behavior equation” should read, “Regarding the rest of the effects in the behavior equation”.*

––––––––––––––––––––––––––––––––––––––––––––––––––––

I revise this sentence on page 13 as the reviewer suggested.

*10. Also on page 13 where you are talking about the SAB models. It would be helpful to have some explanation of how to interpret the following, “Regarding the rest effects in the behavior equation, the rate parameters suggest that adolescents had about 14, 17, 15, 1, and 27 opportunities to change their smoking levels in the five cohorts, respectively.” I am not terribly familiar with these models and I suspect many of your readers will not be as well. What does the rate parameter tell us? Is the difference between 1 and 27 substantial or not.*

––––––––––––––––––––––––––––––––––––––––––––––––––––

I am sorry for the confusion. I now revise it as “the rate parameters are specified as an exponential distribution of waiting times in SAB models, suggesting that the estimated numbers of opportunities for change in smoking levels (i.e., -1 unit, no change, +1 unit) per adolescent between two consecutive time points are about 14, 17, 15, 1, and 27 in the five cohorts, respectively. The rate parameter of 1 for sample dataset 4 indicates that the participants in this cohort were less likely to change their smoking levels than those in the other four cohorts.”

*11. On page 14, you say that you did something by “decomposing the create function and the endowment function” You define these in the methods, but after seeing them applied, you might want to remind readers what they.*

––––––––––––––––––––––––––––––––––––––––––––––––––––

Again, a valid point. The creation function indicates to what extent smokers influence their friends to increase smoking frequency (+1 unit vs. no change), and the endowment function indicates to what extent nonsmokers influence their friends to decrease smoking frequency (-1 unit vs. no change). I now reiterate these details on page 14 in the revised manuscript.

*12.* *Why did you do the analysis for Table 6? What does this add to the paper and how does it help you test your hypotheses?*

––––––––––––––––––––––––––––––––––––––––––––––––––––

Table 6 summarizes the common patterns in findings from the inclusive five sample datasets. As indicated in my reply to comment 13 from reviewer 1, I expect these patterns to be confirmed or challenged by collaboration of scientists across a wide range of disciplines in the near future.

*13. The discussion seems appropriate. In the last paragraph, however, the author makes a statement about policy implication. Designing an intervention to target particular individuals is not policy, it’s an intervention and should called thus.*

––––––––––––––––––––––––––––––––––––––––––––––––––––

I am thankful for a careful reading of the manuscript. I now revise “policy implications” to “preventive implications” in the last paragraph of the “Discussion” section.

**References**

Haas SA, Schaefer DR. With a little help from my friends? Asymmetrical social influence on adolescent smoking initiation and cessation. J Health Soc Behav. 2014; 55: 126–143.

Hagan JF Jr, Shaw JS, Duncan P, editors. Bright Futures: Guidelines for the Health Supervision of Infants, Children, and Adolescents. 3rd ed. Elk Grove Village, IL: American Academy of Pediatrics; 2008.

Hall JA, Valente TW. Adolescent smoking networks: The effects of influence and selection on future smoking. Addict Behav. 2007; 32: 3054–3059.

Handcock MS. Missing Data for of Social Networks. Center for Statistics and the Social Sciences, University of Washington; 2002.

Lakon CM, Hipp JR. On social and cognitive influences: Relating adolescent networks, generalized expectancies, and adolescent smoking. PLoS ONE. 2014; 9: e115668.

Lakon CM, Wang C, Butts CT, Jose R, Timberlake DS, Hipp JR. A dynamic model of adolescent friendship networks, parental influences, and smoking. J Youth Adolesc. 2014; 44: 1767–1786.

Mathys C, Burk WJ, Cillessen AHN. Popularity as moderator of peer selection and socialization of adolescent alcohol, marijuana, and tobacco use. J Res Adolesc. 2013; 23: 513–523.

Montgomery SC, Donnelly M, Bhatnagar P, Carlin A, Kee F, Hunter RF. Peer social network processes and adolescent health behaviors: A systematic review. Prev Med. 2020; 130: 105900.

Osgood DW, Feinberg ME, Ragen DT. Social networks and the diffusion of adolescent problem behavior: Reliable estimates of selection and influence from sixth through ninth grades. Prev Sci. 2015; 16: 832–842.

Schaefer DR, Haas SA, Bishop N. A dynamic model of US adolescents’ smoking and friendship networks. Am J Public Health. 2012; 102: e12–e18.

Shoham DA, Tong L, Lamberson PJ, Auchincloss AH, Zhang J, Dugas L, et al. An actor-based model of social network influence on adolescent body size, screen time, and playing sports. PLoS ONE. 2012; 7: e39795.

US Food and Drug Administration (FDA). Guidance for Industry and FDA Staff: Pediatric Expertise for Advisory Panels. Rockville, MD: US Department of Health and Human Services, Food and Drug Administration, Center for Devices and Radiological Health; 2003. Available from: https://www.fda.gov/media/72451/download.

Wang C, Hipp JR, Butts CT, Jose R, Lakon CM. Coevolution of adolescent friendship networks and smoking and drinking behaviors with consideration of parental influence. Psychol Addict Behav. 2016; 30: 312–324.

Wang C, Hipp JR, Butts CT, Lakon CM. The interdependence of cigarette, alcohol, and marijuana use in the context of school-based social networks. PLoS ONE. 2018; 13: e0200904.
