# Supplementary material for: Smoking prevalence, core/periphery network positions, and peer influence: Findings from five datasets on US adolescents and young adults
Source: PLoS One. 2021 Mar 24;16(3):e0248990. doi: 10.1371/journal.pone.0248990 (PMC7990186; doi:10.1371/journal.pone.0248990)
Supplement: Attachment — Submitted filename: Response to Reviewers.docx [file pone.0248990.s004.docx]

**Response to Reviewers**

PONE-D-20-32056R1

Smoking prevalence, core/periphery network positions, and peer influence: findings from five datasets on US adolescents

PLOS ONE

I would like to thank the editor and reviewer for their very constructive feedback. Incorporating the reviewer’s suggestions for revision has resulted in a greatly improved manuscript. Below, I note the concerns of the reviewer and then explain how I responded to each comment. In all cases, reviewer comments are in italics, while my responses are in plain text.

**Reviewer 2**

*1. I appreciate the citations in the methods about adolescence. However, tobacco control researchers, who will be reading this if it is published, often treat individuals 18-24 as separate from adolescents. It’s fine to include older young adults in this analysis and I understand the NIH and FDA and other organizations treat them as children but your audience will not. This paper will be more credible to your audience if you engage with the norms they use the talk about this population. It would not be hard to reframe the literature review. This would also give you the opportunity to incorporate, more broadly, relevant literature on smoking behavior in your population of interest. You could incorporate this literature and then say that you are going to use the term adolescent but this young adult population is of specific interest in tobacco control and other areas. Please see the following citations:*

*(1) Smoking impacts on prefrontal attentional network function in young adult brains, Francesco Musso, Franziska Bettermann, Goran Vucurevic, Peter Stoeter, Andreas Konrad & Georg Winterer, Psychopharmacology volume 191, pages 159–169(2007)*

*(2) American Journal of Preventive Medicine, Volume 53, Issue 2, August 2017, Pages 139-151, Flavored Tobacco Product Use in Youth and Adults: Findings from the First Wave of the PATH Study (2013–2014)*

*(3) Young Adult Tobacco and E-cigarette Use Transitions: Examining Stability Using Multistate Modeling, Raymond Niaura, PhD, Ilan Rich, Amanda L Johnson, MHS, Andrea C Villanti, PhD, MPH, Alexa R Romberg, PhD, Elizabeth C Hair, PhD, Donna M Vallone, PhD, MPH, David B Abrams, PhD*

*(4) Correlates of Transitions in Tobacco Product Use by U.S. Adult Tobacco Users between 2013–2014 and 2014–2015: Findings from the PATH Study Wave 1 and Wave 2, International Journal of Environmental Research and Public Health*

––––––––––––––––––––––––––––––––––––––––––––––––––––

I thank the reviewer for this suggestion. I now incorporate the line of literature on young adult smokers, as shown in the paragraph on pages 6-7 in the revised manuscript.

“Before proceeding to the next section, we want to note that researchers from various disciplines may have different definitions of the term adolescent. For example, the US Food and Drug Administration (FDA) defines adolescents as individuals in the 12-21 years age group (FDA 2003) and American Academy of Pediatrics (AAP) identifies adolescence as 11 to 21 years of age, including early (ages 11-14 years), middle (ages 15-17 years), and late (ages 18-21 years) adolescence (Hagan et al. 2008). Tobacco control researchers, on the other hand, often treat individuals in the 18-24 years age group as young adults who are separated from adolescents (Musso et al. 2007; Villanti et al. 2017; Kasza et al. 2018; Niaura et al. 2020). Therefore, we incorporate these perspectives from multiple disciplines: we use the term adolescent throughout this study, but we also cover young adult population who are of specific interest in tobacco control and relevant areas.”

In the “Results” section, after introducing the age composition of subjects in the five sample datasets on pages 11-12, I add “the participants in the five sample datasets fit the definitions of adolescents by FDA and AAP (FDA 2003; Hagan et al. 2008), and some of them are also among the young adult population of specific interest in tobacco control and relevant areas (Musso et al. 2007; Villanti et al. 2017; Kasza et al. 2018; Niaura et al. 2020).”

*2. The other reviewer was correct to be concerned about unevenness across the data sets. In the section about sample datasets, it would make sense to highlight the similarities and differences after the datasets are introduced.*

––––––––––––––––––––––––––––––––––––––––––––––––––––

As the reviewer has suggested, I add a paragraph after introducing the datasets to highlight their similarities and differences on pages 8-9 in the revised manuscript:

“All five sample datasets contain the information on their participants’ self-reported personal characteristics (i.e., gender, age, race, parental education level) and smoking levels at two consecutive time points. There are also nonnegligible differences across the datasets. First, the first three sample datasets were collected from high school students during their middle and late adolescent years, while sample datasets 4-5 were collected from college students in late adolescence and emerging adulthood. Second, the NetSense and NetHealth projects used a different strategy from the Add Health study to collect information on friendship networks. The Add Health study used the “name generator”, i.e., a survey question asking each participant to recall the names of his or her friends. Instead, the NetSense and NetHealth projects built friendship networks among their participants with real-word communication events unobtrusively collected by their smartphones. Third, the Zeitgeist toward smoking in 1990s, when the first three sample datasets were collected, was different from that in 2010s, when sample datasets 4-5 were collected.”

*3. For each of the measures, it would be helpful to always have a justification for why the data was treated in the way that it was and likely a citation. For example, why was the cutoff for the highest smoking category 22 days? The doesn’t really make good conceptual sense (e.g. daily smokers may be different than everyone else) and I don’t understand why that was chosen based on the literature.*

––––––––––––––––––––––––––––––––––––––––––––––––––––

This is a great question. I used the same categories as defined in Lakon et al. (2014), Wang et al. (2016), and Wang et al. (2018), and these citations are now added as a justification on page 9 in revised manuscript.

*4. To continue along this line, for the network measures, provide citations for places where these measures have been used in similar research as a way to justify the choice of these measures.*

––––––––––––––––––––––––––––––––––––––––––––––––––––

This is a valid point. Degree centrality and eigenvector centrality have been used as indicators of core-periphery positions in previous research on European tourist online networks (David‑Negre et al. 2018) and international investment networks (Galaso et al. 2020). Betweenness centrality is found to be highly associated with peer influence in crime co-offending networks (Bouchard & Konarski 2014) and online social networks (Johnson et al. 2015). *k*-core has been used as a coreness measure to study HIV risk (Rice et al. 2012; Shahesmaeili et al. 2015) and COVID-19 immunity (Douglas et al. 2020). These citations are now added as a justification of network measures on pages 9-10 in revised manuscript.

*5. I also think the updating that is necessary in the introduction should be followed by changes in the discussion.*

––––––––––––––––––––––––––––––––––––––––––––––––––––

I revise all “adolescent smokers” to “adolescent and young adult smokers” in the “Discussion” section and make updates as the reviewer has suggested.

**References**

Bouchard M, Konarski R. Assessing the core membership of a youth gang from its co-offending network. In: Morselli C, editor. Crime and Networks. New York, NY: Routledge; 2014. pp. 81–96.

David-Negre T, Almedida-Santana A, Hernández JM, Moreno-Gil S. (2018). Understanding European tourists’ use of e-tourism platforms: Analysis of networks. Inf Technol Tour. 2018; 20: 131–152.

Douglas PK, Farahani FV, Douglas DB, Bookheimer, S. (2020). Convalescent blood treatment for COVID-19: Are local donors enough? arXiv 2009.12773 [Preprint]. 2020. Available from: https://arxiv.org/abs/2009.12773.

Galaso P, Sanchez-Diez, A. Core-periphery relations in the international mergers and acquisitions network. Appl Econ Int Dev. 2020; 20: 23–34.

Hagan JF Jr, Shaw JS, Duncan P, editors. Bright Futures: Guidelines for the Health Supervision of Infants, Children, and Adolescents. 3rd ed. Elk Grove Village, IL: American Academy of Pediatrics; 2008.

Johnson SL, Safadi H, Faraj S. The emergence of online community leadership. Inf Syst Res. 2015; 26: 165–187.

Kasza KA, Coleman B, Sharma E, Conway KP, Cummings KM, Goniewicz ML, et al. Correlates of transitions in tobacco product use by US adult tobacco users between 2013–2014 and 2014–2015: Findings from the PATH study wave 1 and wave 2. Int J Environ Res Public Health. 2018; 15: 2556.

Lakon CM, Wang C, Butts CT, Jose R, Timberlake DS, Hipp JR. A dynamic model of adolescent friendship networks, parental influences, and smoking. J Youth Adolesc. 2014; 44: 1767–1786.

Musso F, Bettermann F, Vucurevic G, Stoeter P, Konrad A, Winterer G. Smoking impacts on prefrontal attentional network function in young adult brains. Psychopharmacology. 2007; 191: 159–169.

Niaura R, Rich I, Johnson AL, Villanti AC, Romberg AR, Hair EC, et al. Young adult tobacco and e-cigarette use transitions: Examining stability using multistate modeling. Nicotine Tob Res. 2020; 22: 647–654.

Rice E, Barman-Adhikari A, Milburn NG, Monro W. Position-specific HIV risk in a large network of homeless youths. Am J Public Health. 2012; 102: 141–147.

Shahesmaeili A, Haghdoost AA, Soori H. Network location and risk of human immunodeficiency virus transmission among injecting drug users: Results of multiple membership multilevel modeling of social networks. Addict Health; 2015; 7: 1–13.

US Food and Drug Administration (FDA). Guidance for Industry and FDA Staff: Pediatric Expertise for Advisory Panels. Rockville, MD: US Department of Health and Human Services, Food and Drug Administration, Center for Devices and Radiological Health; 2003. Available from: <https://www.fda.gov/media/72451/download>.

Villanti AC, Johnson AL, Ambrose BK, Cummings KM, Stanton CA, Rose SW, et al. Flavored tobacco product use in youth and adults: Findings from the first wave of the PATH study (2013–2014). Am J Prev Med. 2017; 53: 139–151.

Wang C, Hipp JR, Butts CT, Jose R, Lakon CM. Coevolution of adolescent friendship networks and smoking and drinking behaviors with consideration of parental influence. Psychol Addict Behav. 2016; 30: 312–324.

Wang C, Hipp JR, Butts CT, Lakon CM. The interdependence of cigarette, alcohol, and marijuana use in the context of school-based social networks. PLoS ONE. 2018; 13: e0200904.
