# Supplementary material for: Smoking prevalence, core/periphery network positions, and peer influence: Findings from five datasets on US adolescents and young adults
Source: PLoS One. 2021 Mar 24;16(3):e0248990. doi: 10.1371/journal.pone.0248990 (PMC7990186; doi:10.1371/journal.pone.0248990)
Supplement: Attachment — Submitted filename: Response to Reviewers.docx [file pone.0248990.s005.docx]

**Response to Reviewers**

PONE-D-20-32056R2

Smoking prevalence, core/periphery network positions, and peer influence: findings from five datasets on US adolescents

PLOS ONE

I would like to thank the editor and reviewer for their very constructive feedback. Incorporating the editor and reviewer’s suggestions for revision has resulted in a greatly improved manuscript. Below, I note the concerns of the editor and then explain how I responded to each comment. In all cases, comments from the editor are in italics, while my responses are in plain text.

**Editor**

*Please revise the paper to make it clear that you are studying young adults and not adolescents. There has been a move at NIH, for example, to stop designating people 18-21 as children. The way that study population is studies will make your paper hard for people to use in the field.*

––––––––––––––––––––––––––––––––––––––––––––––––––––

I thank the editor for this suggestion. In the revised manuscript I completely adopt the tobacco control researchers’ approach by defining individuals in the 18-24 years age group as young adults who are separated from adolescents aged 12-17. The participants from the sample datasets 4 and 5 had an average age of 18.28 and 18.01, respectively, and thus fit this definition. However, the participants from the first three sample datasets had an average age of 15.53, 15.95, and 14.19, respectively. The readers will be utterly confused if I call them young adults as well. As a result, in the revised manuscript I clarify I am studying both adolescents and young adults, and corresponding changes are made in the title, abstract, and manuscript.
